# Highly multiplexed digital PCR assay for simultaneous quantification of variant allele frequencies and copy number alterations of *KRAS* and *GNAS* in pancreatic cancer precursors

**DOI:** 10.1002/1878-0261.70011

**Published:** 2025-03-12

**Authors:** Junko Tanaka, Tatsuo Nakagawa, Yusuke Ono, Yoshio Kamura, Takeshi Ishida, Hidemasa Kawabata, Kenji Takahashi, Hiroki Sato, Andrew S. Liss, Yusuke Mizukami, Takahide Yokoi

**Affiliations:** ^1^ Center for Digital Services – Healthcare Research & Development Group, Hitachi, Ltd. Tokyo Japan; ^2^ Institute of Biomedical Research Sapporo Higashi Tokushukai Hospital Japan; ^3^ Department of Advanced Genomic Community Healthcare Asahikawa Medical University Japan; ^4^ Division of Gastroenterology, Department of Medicine Asahikawa Medical University Japan; ^5^ Division of Gastrointestinal and Oncologic Surgery Massachusetts General Hospital and Harvard Medical School Boston MA USA

**Keywords:** copy number alterations, digital PCR, melting curve analysis, multiplex, pancreatic cancer, variant allele frequency

## Abstract

Pancreatic intraepithelial neoplasia (PanIN) and intraductal papillary mucinous neoplasms (IPMNs) are pancreatic ductal adenocarcinoma (PDAC) precursor lesions. Detecting these precursors and monitoring their progression are crucial for early PDAC diagnosis. Digital PCR (dPCR) is a highly sensitive nucleic acid quantification technique and offers a cost‐effective option for patient follow‐up. However, the clinical utility of conventional dPCR is restricted by multiplexing constraints, particularly due to the challenge of simultaneously quantifying multiple mutations and amplifications. In this study, we applied highly multiplexed dPCR and melting curve analysis to simultaneously measure single nucleotide mutations and amplifications of *KRAS* and *GNAS*. The developed 14‐plex assay included both wild‐type and mutant *KRAS*, a common driver gene in both PanIN and IPMN, and *GNAS*, which is specifically mutated in IPMN, along with *RPP30*, a reference gene for copy number alterations (CNAs). This multiplex dPCR method detected all target mutations with a limit of detection below 0.2% while quantifying CNAs. Additionally, the assay accurately quantified variant allele frequencies in liquid biopsy and tissue samples from both pancreatic neoplasm precursor and PDAC patients, indicating its potential for use in comprehensive patient follow‐up.

## Graphical abstract

Combining melting curve analysis enhances the multiplexing capability of digital PCR. Here, we developed a 14‐plex assay to simultaneously measure single nucleotide mutations and amplifications of *KRAS* and *GNAS*, which are common driver genes in pancreatic cancer precursors. This assay accurately quantified variant allele frequencies in clinical samples from both pancreatic neoplasm precursor and PDAC patients.

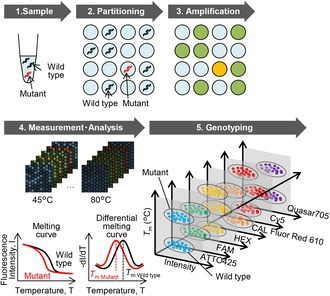

**Abbreviations**
cfDNA: cell‐free DNA
CNA: copy number alteration
dPCR: digital PCR
ERCP: endoscopic retrograde cholangiopancreatography
FNA/B: fine‐needle aspiration/biopsy
FPE: formalin‐fixed, paraffin‐embedded
FWHM: full width at half maximum
IPMN: intraductal papillary mucinous neoplasm
LOD: limit of detection
NGS: next‐generation sequencing
PanIN: pancreatic intraepithelial neoplasia
PDAC: pancreatic ductal adenocarcinoma
*T*_m_
: melting temperature
VAF: variant allele frequency

## 1 Introduction

Pancreatic ductal adenocarcinoma (PDAC) is a highly fatal disease with a 5‐year survival rate of approximately 10% [1], and it is projected to become the second leading cause of cancer death in the United States by 2040 [2]. The poor prognosis of PDAC is due primarily to the fact that more than 80% of cases are diagnosed at an unresectable stage [3]. Consequently, there is an urgent need for effective screening techniques to identify high‐risk patients and detect PDAC at an early, surgically resectable stage to significantly improve clinical outcomes.

Pancreatic intraepithelial neoplasia (PanIN) and intraductal papillary mucinous neoplasms (IPMNs) have been identified as precursor lesions of PDAC [4, 5]. PanINs are the most common precursors of PDAC; however, owing to their typical microscopic morphology, these lesions are often undetectable despite their widespread distribution, even in individuals without clinically notable pancreatic neoplasms [6]. In contrast, IPMNs are visible due to cystic formation associated with abundant mucin production. While IPMNs are often benign, a significant proportion progress to malignancy; the incidence of pancreatic malignancy 15 years after IPMN diagnosis is 15.0% [7]. Unfortunately, 32% of patients with pancreatic carcinoma already have advanced cancer at diagnosis [7], and early detection of the transition from IPMN to invasive cancer remains challenging.

*KRAS* mutations are the most frequently detected genetic alterations in these precursors; they occur early in the carcinogenic process and are found in more than 90% of PDAC tumours [8]. *GNAS* mutations are recurrently present in IPMNs at frequencies ranging from 40% to 75% [4]. Gene amplification can also serve as a biomarker for these neoplasms, with *KRAS* and *GNAS* amplifications being present in 1.5% and 1.1% of PDAC patients, respectively, according to the ICGC/QCMG/TCGA/UTSW/CPTAC cohort dataset of pancreatic cancer (cBioPortal for Cancer Genomics, http://www.cbioportal.org, last accessed July 31, 2024). A clinical hallmark of IPMN is the presence of multiple lesions, each of which is molecularly distinct [9, 10]. The diverse *KRAS* and *GNAS* mutations in the multiple lesions of IPMN clonally evolve into invasive carcinoma [11, 12]. Therefore, capturing these tumour‐causing driver mutations and monitoring the variation in mutant abundance and changes in genotype balance will facilitate early detection of pancreatic carcinoma.

Next‐generation sequencing (NGS) and digital PCR (dPCR) have enabled the profiling of genetic alterations in DNA from liquid biopsy samples or minimal amounts of tissue collected by fine‐needle aspiration/biopsy (FNA/B) [13, 14, 15, 16]. Compared with NGS, dPCR is more cost‐effective and has a fast turnaround time, thereby providing a powerful tool for monitoring cancers, such as the carcinogenesis of IPMN. However, the clinical utility of conventional dPCR remains limited owing to its multiplexing constraints, which prevent the quantification of all actionable biomarkers in a single assay. In particular, few reports exist in which dPCR was used to quantify single nucleotide mutations and gene amplifications simultaneously. In addition to single nucleotide mutations, CNA is recognised as a valuable biomarker for cancer monitoring. Studies have shown that the CNA status of DNA in tumour correlates with that in cell‐free DNA (cfDNA) in plasma, and that patients with CNA‐positive cfDNA in plasma tend to have a poorer prognosis [17, 18, 19]. Previous reports detected only one or a few types of single nucleotide mutations alongside gene amplification [20, 21], with limited multiplexing capabilities. Several methods have been proposed to increase dPCR multiplicity, including amplification curve analysis [22] and amplitude modulation [15, 16, 23]. However, in highly multiplexed dPCR, genotyping is challenging when different genotypes are present in the same compartment, requiring techniques to detect only the mutant without detecting the wild type [23] or reducing the amount of DNA to avoid double‐positive wells of the wild type and mutant [16]. Copy number alteration (CNA) quantification requires the detection of reference genes in addition to target genes, making it unsuitable for evaluation with these techniques.

In this report, we demonstrated the use of highly multiplexed dPCR combined with melting curve analysis to simultaneously measure single nucleotide mutations and gene amplification of *KRAS* and *GNAS* for precise analysis of samples from IPMN and PDAC patients. We selected the eight most common mutations (G12D, G12R, G12V, G13D, G12A, G12C, G12S and Q61H) in codons 12, 13 and 61 of *KRAS*; the two most common mutations (R201H and R201C) in codon 201 of *GNAS*; and *RPP30* as the reference gene for evaluating CNAs. This 14‐plex dPCR assay was developed to amplify amplicons of approximately the same size for multiple targets, enabling accurate CNA quantification of fragmented DNA samples. Probes for each of the 14 genotypes were designed to avoid overlapping probe colours or melting temperature (*T*_m_) values, allowing accurate genotyping of wells containing multiple genotypes. This 14‐plex dPCR was used to measure genomic DNA derived from cultured cells, confirming accurate genotyping and CNA quantification. Furthermore, DNA extracted from liquid biopsy samples (plasma, duodenal fluid and pancreatic juice) and tissue samples obtained from IPMN and PDAC patients was analysed, and mutation frequencies correlated well with results obtained via other gene quantification techniques. Some patient samples showed potential *GNAS* gene amplification without *GNAS* mutations. To our knowledge, this is the first demonstration of an assay that combines dPCR and six‐colour melting curve analysis to discriminate more than 10 genotypes simultaneously and to quantify gene amplification.

## 2 Methods

### 2.1 Patients and samples

The study involved patients with PDAC or IPMN diagnosed at Asahikawa Medical University, Japanese Red Cross Asahikawa Hospital, Asahikawa Kosei Hospital, Teine Keijinkai Hospital, Shibetsu City Hospital, and Sapporo Higashi Tokushukai Hospital. Thirteen plasma samples; 10 duodenal fluid samples; 10 pancreatic juice samples and 10 formalin‐fixed, paraffin‐embedded (FFPE) tissue samples were collected (between June 2017 and February 2024) and evaluated in this study. Blood samples were collected in 10‐mL PAXgene Blood ccfDNA Tubes (BD, Franklin Lakes, NJ, USA) and gently inverted. Similarly, all blood samples were transported to the laboratory at Sapporo Higashi Tokushukai Hospital at 4 °C. Cell‐free plasma was prepared by subjecting the blood samples to two centrifugation steps: centrifugation at 1900 ***g*** for 15 min and transfer to new 15 mL tubes, followed by another centrifugation at 1900 ***g*** for 10 min at 20–25 °C. Finally, the supernatant was collected as the plasma fraction. Duodenal fluid samples (3–5 mL) were directly aspirated during endoscopy via an MMI tracheal suction kit (Muranaka Medical Instruments, Osaka, Japan). The samples were subsequently mixed with TE buffer (100 mm Tris–HCl, 50 mm EDTA pH 8.0) to a final concentration. The samples from all the hospitals were then shipped to the genomics laboratory at Sapporo Higashi Tokushukai Hospital via refrigerated transport at 4 °C. Fresh pancreatic juice samples (1 mL; sample less than 1 mL were adjusted to 1 mL with PBS) were collected via the endoscopic retrograde cholangiopancreatography (ERCP) catheter and frozen immediately after the procedure (within 10 min). For pancreatic tissue DNA, unstained sections from FFPE samples (10 μm thick) were used for genomic DNA extraction.

This study was conducted in compliance with the guidelines of the Helsinki Declaration and the Ethical Guidelines for Medical and Health Research Involving Human Subjects issued by the Japanese government. The experimental protocols were approved by the ethics committees of Hitachi, Ltd. (303‐1) and Asahikawa Medical University (C21138). Written informed consent was obtained from all patients. The methods were carried out in accordance with the approved guidelines.

*KRAS* mutant genomic DNA reference standards (Horizon Diagnostics, Cambridge, UK); *GNAS* mutant genomic DNA (950‐5‐BIK [24], provided by the MGH Pancreatic Tumor Bank); and genomic DNA from the cell lines listed in Table S1 were also used as DNA templates for dPCR. Before use, all the genomic DNA reference standards and genomic DNA from the cell lines were sheared to an average size of 200 bp via a Covaris M220 focused ultrasonicator (Covaris, Woburn, MA, USA).

### 2.2 Extraction and quantification of DNA

Plasma cell‐free DNA (cfDNA) and pancreatic juice‐derived DNA were isolated via the MagDEA Dx MV II (Precision System Science, Chiba, Japan) of the MagLEAD automatic DNA purification system (Precision System Science). One millilitre of plasma and pancreatic juice was processed per purification run and DNA was eluted with 100 μL of elution buffer. Purified DNA from all runs of each sample was pooled and concentrated to 100 μL using DNA Clean & Concentrator‐5 kit (Zymo Research, Irvine, CA, USA) according to the manufacturer's instructions. DNA from the duodenal fluid was extracted via the QIAamp Min Elute ccfDNA Midi Kit (Qiagen, Hilden, Germany) according to the manufacturer's instructions. Purified liquid‐derived DNA was eluted with 100 μL of elution buffer. FFPE‐derived DNA was isolated with the GeneRead DNA FFPE Kit (Qiagen) or the QIAamp DNA FFPE Advanced UNG Kit (Qiagen) and eluted in 30 μL of elution buffer. The purified DNA was quantified via a Qubit dsDNA HS Assay Kit on a Qubit4 fluorometer (Thermo Fisher Scientific, Waltham, MA, USA) and stored at −80 °C until analysis. In this study, plasma samples with a cfDNA concentration of ≥ 10 ng·mL^−1^ were selected and used for analysis.

### 2.3 Quantification of *KRAS* , *GNAS* and *RPP30* by multiplex dPCR combined with melting curve analysis

Absolute quantification of *KRAS* and *GNAS* mutations was carried out via dPCR combined with melting curve analysis. The sequences of the primers and probes are listed in Table S2. The composition of the reaction solution used for performing PCR and measuring *T*_m_ in the 14‐plex assay in the wells was as follows: 1× QuantStudio 3D Digital PCR Master Mix v2 (Thermo Fisher Scientific), 0.25 μm *KRAS* forward primer, 2.0 μm *KRAS* reverse primer, 0.25 μm *GNAS* forward primer, 2.0 μm *GNAS* reverse primer, 0.25 μm *RPP30* forward primer, 2.0 μm *RPP30* reverse primer, 0.5 μm *KRAS* wild‐type detection probe, 0.5 μm *KRAS* G12R detection probe, 0.5 μm *KRAS* G12D detection probe, 0.5 μm *KRAS* G12V detection probe, 0.5 μm *KRAS* G12A detection probe, 0.5 μm *KRAS* G13D detection probe, 0.5 μm *KRAS* G12S detection probe, 0.5 μm *KRAS* G12C detection probe, 0.5 μm *KRAS* pseudogene blocker, 0.5 μm *KRAS* Q61H detection probe, 0.5 μm *GNAS* wild‐type detection probe, 0.5 μm *GNAS* R201H detection probe, 0.5 μm *GNAS* R201C detection probe, 0.5 μm *RPP30* wild‐type detection probe, 0.5 m betaine and 30 ng of clinical sample solution or 4 μL of clinical sample solution in a final reaction volume of 15 μL.

After the addition of 14.5 μL of PCR mixture to a QuantStudio 3D Digital PCR 20K Chip (Thermo Fisher Scientific), PCR was performed with a thermal cycler. Amplification was carried out as follows: 10 min at 96 °C and 60 cycles of 30 s at 98 °C and 2 min at 50 °C. After PCR, the chips were slowly cooled from 98 to 10 °C at a ramp rate of 0.3 °C·s^−1^. Then, the chip was placed on the temperature control stage of the developed *T*_m_ measuring device, and six‐colour fluorescence images of the chip were acquired while the temperature was increased from 45 °C to 80 °C at 2.0 °C·min^−1^. Melting curve analysis of each well was performed from the obtained six‐colour fluorescence images of the chip, and the *T*_m_ value was calculated according to a previous report [25]. The number of positive wells containing the target DNA was determined on the basis of the fluorescence intensity and *T*_m_ value. The concentration of the target DNA in the clinical sample was calculated from the number of positive wells using the Poisson's statistics.

### 2.4 Mutation profiling by singleplex dPCR or targeted amplicon sequencing

For the validation of mutation profiles, droplet dPCR on the Bio‐Rad platform or targeted amplicon sequencing was conducted via previously reported methods [16].

Using a QX200 Droplet Digital PCR System (Bio‐Rad, Hercules, CA, USA), we performed droplet dPCR analysis. Custom probes and primers previously designed for five major PDAC‐related mutations in *KRAS* codon 12 and *GNAS* codon 201 were used [16]. Encapsulated droplet PCR was performed under the previously described reaction conditions [16]. The absolute copy number input and ratio of mutated fragments were calculated via quantasoft analysis pro software (ver 1.0; Bio‐Rad) on the basis of the Poisson distribution. The samples were scored as positive for cfDNA when at least three mutant droplets were detected via dPCR.

The targeted amplicon sequence was performed as follows. Briefly, the pancreatic cancer‐associated custom DNA sequencing panel, which we previously designed, was employed for the mutation profiling of eight genes, including the *KRAS* G12/G13 and *GNAS* R201 hotspot regions, as reported previously [16]. 10–60 ng of DNA was amplified via this panel, and sequencing libraries were subsequently constructed. Sequencing and data analysis were performed via the Ion GeneStudio S5 system (Thermo Fisher Scientific) as previously described [16].

### 2.5 Quantification of the CNAs of *KRAS* , *GNAS* and *RPP30* by conventional dPCR

Another absolute quantification for the CNAs of *KRAS*, *GNAS* and *RPP30* was carried out via the QIAcuity Digital PCR System (Qiagen) and QIAcuity Nanoplate 8.5k 24‐well (Qiagen). Copy number ratios of *KRAS* to *RPP30* and *GNAS* to *RPP30* were measured separately via dPCR assays. The primers and probes for *KRAS*, *GNAS* and *RPP30* were commercially available and well validated (assay ID for *KRAS*: dHsaCP1000033; assay ID for *GNAS*: dHsaCP1000398; assay ID for *RPP30*: dHsaCP2500350; Bio‐Rad). The composition of the reaction mixture used for dPCR was as follows: 1× Probe PCR Master Mix (Qiagen), 1× mixture of primers and probes (Bio‐Rad) and 10 000 copies of genomic DNA from the cell lines in a final reaction volume of 12 μL. Partitioning, thermal cycling and signal detection were performed according to the manufacturer's instructions. The data were analysed via the qiacuity software suite (Qiagen).

## 3 Results

### 3.1 Overall workflow of dPCR combined with melting curve analysis

**Fig. 1:**
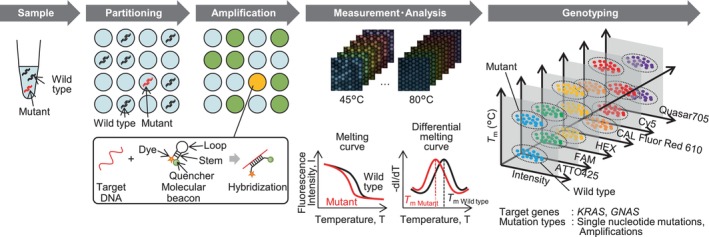
Procedure of dPCR combined with the melting curve assay. Samples including target DNA were partitioned into microwells and asymmetric PCR was performed with molecular beacons in the wells. Fluorescence images depending on temperature were captured with six‐colour filters and the melting curves were analysed for each well. Genotyping was performed according to fluorescence intensity, dye colour and *T*_m_. dPCR, digital PCR; *T*_m_, melting temperature.

We developed a dPCR measurement system combined with melting curve analysis. Figure 1 shows the overall workflow of the system. The dPCR measurement with melting curve analysis consisted of five steps: preparation of the reaction mixture, partitioning of the reaction solution into microwells, PCR, fluorescence measurement and melting curve analysis and genotyping. First, in the reaction solution preparation step, the clinical sample, polymerases, primers and probes are mixed. For the probe, a molecular beacon is used [26], which is not degraded by polymerase during PCR. The free molecular beacon has a stem–loop structure, as shown in Fig. 1. The loop moiety is complementary to the target DNA, and a fluorescent dye and a quencher are bound to each end of the stem. In the partitioning step, the reaction mixture is partitioned into a silicon chip with up to 2 × 10^4^ wells. In the PCR step, the target DNA in the wells is amplified via asymmetric PCR to obtain single‐stranded amplicons complementary to the molecular beacon probe [27, 28]. If the wells contain target DNA (referred to as ‘positive wells’), the molecular beacons hybridise to the amplified target, and the fluorescence intensity of the wells becomes greater than that of wells without the target DNA (referred to as ‘negative wells’). In the fluorescence measurement and melting curve analysis step, the chip is placed on the temperature control stage of the custom‐made instrument, and fluorescence images are captured while the temperature of the chip is controlled. The instrument has been expanded from the previously reported four‐colour channel [25] to a six‐colour channel. The melting curve of each well is obtained by plotting the fluorescence intensity against the temperature of the chip, and the *T*_m_ is calculated by differentiating the melting curve. Finally, in the genotyping step, the genotype of the DNA in the wells is determined from the fluorescence intensity, colour of the probe dye and *T*_m_.

### 3.2 Genotyping of *KRAS* and *GNAS*

We extended and improved a previously reported assay [25, 29] to quantify variant allele frequencies (VAFs) and evaluate the CNAs of the driver genes for pancreatic cancer precursors, *KRAS* and *GNAS*, for the following 14 genotypes. We selected the eight most common *KRAS* mutations (G12D, G12R, G12V, G13D, G12A, G12C, G12S and Q61H) and their wild‐type counterparts at codons 12/13 and 61, the two most common *GNAS* mutations (R201H and R201C) and the wild‐type allele. Additionally, wild‐type *RPP30* was used as a reference gene to evaluate CNAs in *KRAS* and *GNAS*.

**Fig. 2:**
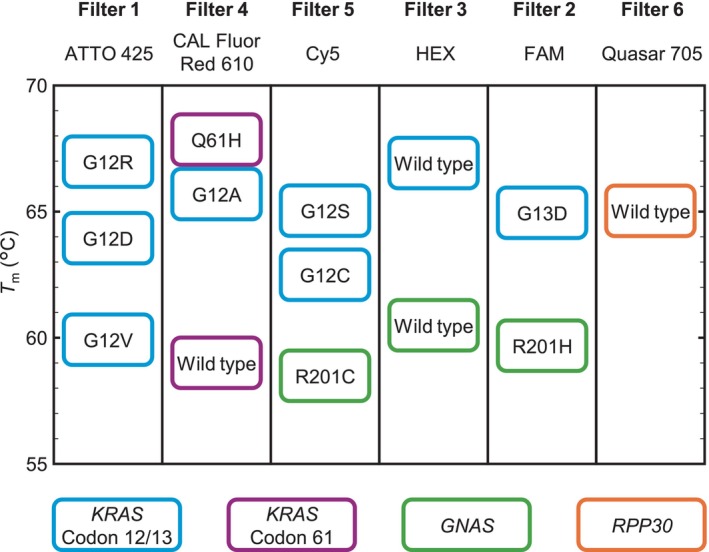
Layout for genotyping wild‐type and mutant *KRAS*, *GNAS* and *RPP30* strains via multiplex dPCR combined with melting curve analysis. The horizontal axis represents the fluorescence intensities measured with the ATTO 425, FAM, HEX, CAL Fluor Red 610, Cy5 and Quasar 705 filters, and the vertical axis represents the *T*_m_ value. Blue, *KRAS* codon 12/13; purple, *KRAS* codon 61; green, *GNAS*; orange, *RPP30*. *T*_m_, melting temperature.

Figure 2 shows the layout for genotyping wild‐type and mutant *KRAS*, *GNAS* and *RPP30* by multiplex dPCR combined with melting curve analysis. The horizontal axis represents the fluorescence intensities measured with the ATTO 425, FAM, HEX, CAL Fluor Red 610, Cy5 and Quasar 705 filters, and the vertical axis represents the *T*_m_ value. All the genotypes are arranged to have different fluorescent dye colours or *T*_m_ values, which can be controlled by the probe sequence and length.

Table 1 and Figs S1‐1, S1‐2 and S2 show the genotyping results for various genomic DNA standards. The detected DNA groups were clearly clustered on the basis of fluorescence intensity and *T*_m_ values. In dPCR combined with melting curve analysis, the type of target DNA in a well can be distinguished by the colour of the fluorescent dye, the *T*_m_ value and the full width at half maximum (FWHM) of the differential melting curve, even if multiple types of target DNA are contained in a single well. For example, wild‐type *KRAS* and wild‐type *GNAS* are detected with the same fluorescent dye. On the basis of the *T*_m_ value and the FWHM of the differential melting curve, the single positive well of the wild‐type *KRAS* and wild‐type *GNAS* and the double positive well of both can be discriminated. The genotyping window for counting was manually chosen on the basis of the results of the genomic DNA standards in Figs S1‐1, S1‐2 and S2, and the same window was used for all subsequent experiments. Table 1 shows the results of discriminating and counting each genotype from Figs S1‐1, S1‐2 and S2, and the genotyping results were consistent with the input samples.

**Table 1:** Genotyping results of genomic DNA measured by dPCR combined with melting curve analysis. dPCR, digital PCR; WT, wild type.

Description of genomic DNA	*KRAS*, codon 12/13								*KRAS*, codon 61		*GNAS*, codon 201		
	WT	G12R	G12D	G12V	G12A	G13D	G12S	G12C	WT	Q61H	WT	R201H	R201C
WT	100.0%	0.0%	0.0%	0.0%	0.0%	0.0%	0.0%	0.0%	100.0%	0.0%	100.0%	0.0%	0.0%
KRAS 50% G12R	49.5%	50.2%	0.2%	0.1%	0.0%	0.0%	0.0%	0.1%	100.0%	0.0%	99.9%	0.1%	0.0%
KRAS 50% G12D	49.1%	0.0%	50.9%	0.0%	0.0%	0.0%	0.0%	0.0%	100.0%	0.0%	99.9%	0.1%	0.1%
KRAS 50% G12V	49.4%	0.0%	0.0%	50.5%	0.1%	0.1%	0.0%	0.0%	100.0%	0.0%	100.0%	0.0%	0.0%
KRAS 50% G12A	49.1%	0.0%	0.0%	0.1%	50.8%	0.0%	0.0%	0.0%	100.0%	0.0%	99.9%	0.0%	0.1%
KRAS 50% G13D	51.1%	0.0%	0.0%	0.0%	0.0%	48.8%	0.0%	0.1%	100.0%	0.0%	99.9%	0.1%	0.0%
KRAS 50% G12S	52.2%	0.0%	0.0%	0.0%	0.0%	0.0%	47.4%	0.4%	100.0%	0.0%	99.9%	0.0%	0.1%
KRAS 50% G12C	50.1%	0.2%	0.0%	0.0%	0.0%	0.0%	0.0%	49.6%	100.0%	0.0%	99.4%	0.1%	0.5%
KRAS 5% G12D, 25% G13D, 5% G12C, 5% Q61H	67.7%	0.0%	4.6%	0.0%	0.0%	23.9%	0.0%	3.8%	93.8%	6.2%	99.8%	0.2%	0.1%
KRAS 65% G12V, GNAS 50% R201H	31.3%	0.0%	0.0%	68.6%	0.1%	0.0%	0.0%	0.0%	100.0%	0.0%	50.0%	50.0%	0.0%
GNAS 16.3% R201C	99.9%	0.0%	0.0%	0.0%	0.0%	0.1%	0.0%	0.0%	100.0%	0.0%	81.7%	0.0%	18.2%

### 3.3 Validation of the limit of detection

We then evaluated the linearity and sensitivity of multiplex dPCR combined with curve analysis for *KRAS* and *GNAS* detection. Fragmented wild‐type genomic DNA standards spiked with the respective fragmented genomic DNA standards were used for linearity evaluation. The linearity of quantification of *GNAS* R201C was specifically evaluated via the use of fragmented wild‐type genomic DNA standards spiked with DNA extracted from FFPE samples containing the R201C mutation. As shown in Fig. S3, the input and detection ratios were proportional in all samples, with *R* ^2^ correlation coefficients above 0.96.

The limit of detection (LOD) for each mutant was calculated by adding threefold standard deviation to the mean value measured via a genomic DNA standard consisting of 100% wild‐type standard (*N* = 8). Table 2 shows the LODs of each *KRAS* and *GNAS* mutant. For all mutations, the LOD was lower than 0.2%. These LOD values were comparable to those of other multiplex dPCR assays [30, 31].

**Table 2:** LOD of each *KRAS* and *GNAS* mutant measured by dPCR combined with melting curve analysis. dPCR, digital PCR; LOD, limit of detection.

Gene	Mutant	LOD (%)
*KRAS*	G12R	< 0.01
*KRAS*	G12D	0.08
*KRAS*	G12V	0.03
*KRAS*	G12A	0.01
*KRAS*	G13D	0.10
*KRAS*	G12S	0.05
*KRAS*	G12C	0.11
*KRAS*	Q61H	< 0.01
*GNAS*	R201H	0.11
*GNAS*	R201C	0.09

### 3.4 CNA quantification

For accurate CNA quantification, adjusting the amplicon sizes of the target and reference genes to approximately the same length is crucial. Initially, we used primer sequences from previous reports for multiplex dPCR, resulting in different amplicon sizes for the target and reference genes: 98 bp for *KRAS*, 96 bp for *GNAS* and 65 bp for *RPP30* [16, 25, 29, 32]. Genomic DNA from cultured SW48 cells, which have no gene amplification of *KRAS* or *GNAS*, was fragmented and quantified. The copy number ratio of *KRAS* or *GNAS* to *RPP30* was 0.77 ± 0.07 or 0.75 ± 0.07, respectively, for genomic DNA fragmented to cfDNA size, whereas it was 1 for non‐fragmented genomic DNA. This variation occurs because the detection rate is greater for genes with smaller amplicon sizes when fragmented genomic DNA is measured [29]. The fragment size distribution differs between cfDNA in plasma and that in urine [33]. Furthermore, the fragment size of cfDNA from cancer patients is smaller than that of cfDNA from healthy individuals [34]. Hence, to compare CNAs regardless of the disease and sample type, the amplicon sizes of the target and reference genes must be adjusted to approximately the same length. We therefore redesigned the primers for *RPP30*, adjusting the amplicon sizes of *KRAS*, *GNAS* and *RPP30* in multiplex dPCR combined with melting curve analysis to 98 bp, 96 bp and 98 bp, respectively. We quantified fragmented genomic DNA from cultured SW48 cells via multiplex dPCR combined with melting curve analysis using redesigned primers. The copy number ratios of *KRAS* and *GNAS* to *RPP30* were 0.93 ± 0.01 and 0.99 ± 0.03, respectively, which were close to 1, similar to those measured for non‐fragmented genomic DNA.

**Fig. 3:**
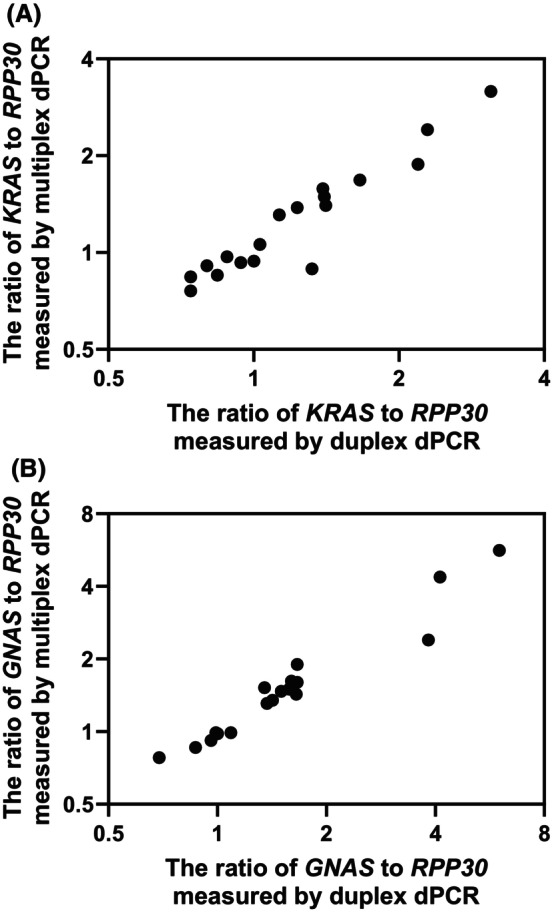
Results of CNA quantification by multiplex dPCR combined with melting curve analysis compared with those of conventional duplex dPCR via a commercially available primer/probe mixture for CNA evaluation. Genomic DNA extracted from the 18 cell lines was measured via multiplex dPCR combined with melting curve analysis, and the total copy number of the wild‐type and mutant forms of *KRAS* or *GNAS* and the copy number of the reference gene, *RPP30*, were determined. Conventional duplex dPCR is able to measure only the total copy number of *KRAS*, *GNAS* and *RPP30*, and the genotype of mutant forms cannot be identified. On the basis of the count results, the ratio of the total copy number of *KRAS* or *GNAS* to the copy number of the reference gene *RPP30* was calculated. (A) The ratio of *KRAS* to *RPP30* (*R* ^2^ = 0.94); (B) the ratio of *GNAS* to *RPP30* (*R* ^2^ = 0.93). CNA, copy number alteration; dPCR, digital PCR.

CNA quantification was then verified through the use of genomic DNA extracted from the 18 cell lines listed in Table S1. Genomic DNA was measured via multiplex dPCR combined with melting curve analysis, and the total copy number of the wild‐type and mutant forms of *KRAS* or *GNAS* and the copy number of the reference gene, *RPP30*, were determined. On the basis of the count results, the ratio of the total copy number of *KRAS* or *GNAS* to the copy number of the reference gene *RPP30* was calculated. Figure 3 shows a comparison of CNA quantification results obtained by the multiplex dPCR assay with those obtained by conventional duplex dPCR using a commercially available primer/probe mixture for CNA evaluation. The copy number ratios of *KRAS* or *GNAS* to *RPP30* obtained from multiplex dPCR combined with melting curve analysis correlated well with those obtained from conventional duplex dPCR, with *R* ^2^ correlation coefficients of 0.94 and 0.93 respectively. These results indicate that multiplex dPCR combined with melting curve analysis maintains high CNA quantification accuracy even when the number of multiplexes exceeds 10.

### 3.5 Quantitative performance in clinical samples

To determine the feasibility of genotyping and CNA quantification of tumour DNA directly from the clinical samples of IPMN and PDAC patients, we performed multiplex dPCR on four different clinical materials: plasma, duodenal fluid, pancreatic juice and tumour tissue (FFPE). We selected three liquid samples for assessing IPMN patients and PDAC patients preoperatively and for confirming the presence of tumour DNA after pancreatectomy. Plasma is suitable for repeat testing because of its less invasive collection, though the DNA yield is small. Obtaining duodenal fluid and pancreatic juice samples is more invasive, as these samples are collected via endoscopy and catheter insertion, but they are collected from close proximity to the pancreas, yielding a greater percentage of tumour‐derived DNA. After DNA extraction from each clinical sample and subsequent purification, the DNA concentration was quantified via a fluorescent reagent for nucleic acid quantification, and dPCR was performed through the use of 30 ng of DNA (equivalent to 9000 copies) as the template. If the DNA concentration of the sample was low, 4 μL of DNA (the maximum allowable amount of template in the reaction mixture) was added.

**Fig. 4:**
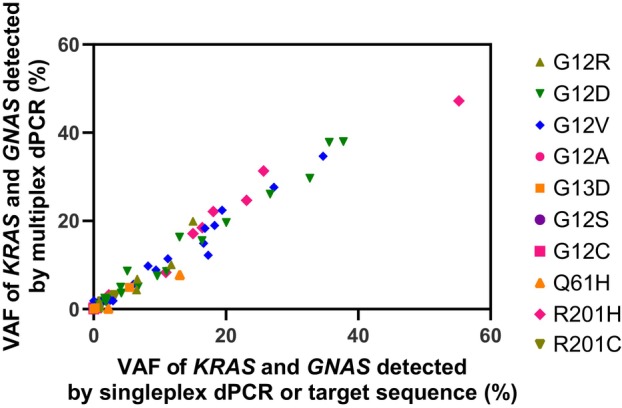
Comparison of *KRAS* and *GNAS* VAFs detected by multiplex dPCR combined with melting curve analysis with *KRAS* and *GNAS* VAFs detected via conventional singleplex dPCR or targeted sequencing. VAFs of *KRAS* and *GNAS* mutations were measured in 43 clinical samples, including plasma, duodenal fluid, pancreatic juice and tumour tissue. The colours and shapes of the plots show the genotype observed by multiplex dPCR using melting curve analysis. The *R* ^2^ correlation coefficient was > 0.98. VAFs, variant allele frequencies; dPCR, digital PCR.

Multiplex dPCR combined with melting curve analysis accurately detected *KRAS* and *GNAS* mutations in clinical samples, with VAFs comparable to those obtained from conventional singleplex dPCR data or targeted sequencing. In conventional singleplex dPCR, each variant was measured in a separate assay. Figure 4 shows the comparison of VAFs of *KRAS* and *GNAS* mutations detected in 43 clinical samples, including plasma, duodenal fluid, pancreatic juice and tumour tissue. Detailed comparisons of the VAFs of *KRAS* and *GNAS* mutations for each clinical sample type are shown in Fig. S4. VAFs of *KRAS* and *GNAS* detected by multiplex dPCR correlated well with VAFs detected by conventional singleplex dPCR or targeted sequencing (*R* ^2^ > 0.98). These results indicate that multiplex dPCR combined with melting curve analysis can accurately quantify VAFs regardless of the origin of the clinical sample.

**Fig. 5:**
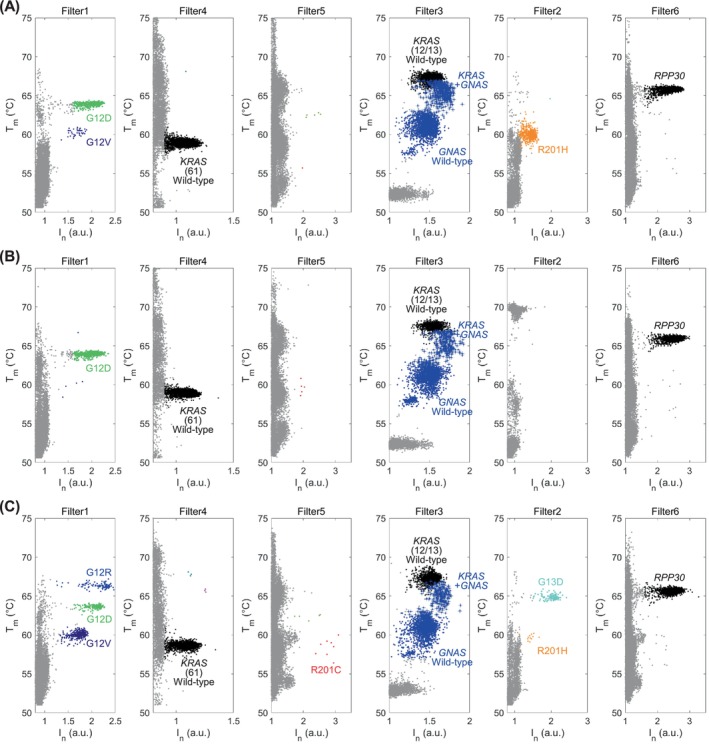
Examples of genotyping results of IPMN and PDAC patients. The horizontal axis represents the fluorescence intensities measured by each filter, and the vertical axis represents the *T*_m_ value. Wild‐type and mutant *KRAS*, *GNAS* and *RPP30* were genotyped by the fluorescence intensity, colour of the probe dye and *T*_m_. (A) Pancreatic juice sample #6 (IPMN patient); (B) pancreatic juice sample #2 (PDAC patient); (C) pancreatic juice sample #8 (PDAC patient). *I*_n_, normalised intensity; IPMN, intraductal papillary mucinous neoplasm; PDAC, pancreatic ductal adenocarcinoma; *T*_m_, melting temperature.

Representative genotyping results for pancreatic juice samples obtained from IPMN and PDAC patients are shown in Fig. 5. As shown in Fig. 5A, G12D and G12V of *KRAS* and R201H of *GNAS* were detected simultaneously, with VAFs of 15.5%, 1.2% and 17.1% respectively. As shown in Fig. 5B, G12D of *KRAS* was detected, with a VAF of 38.0%. As shown in Fig. 5C, G12R, G12D, G12V and G13D of *KRAS* and R201H and R201C of *GNAS* were detected simultaneously, with VAFs of 4.4%, 7.5%, 14.9%, 4.9%, 0.5% and 0.3% respectively. Each VAF was nearly identical to those measured by the other gene quantification methods. The simultaneous detection of multiple mutations in Fig. 5A suggests that IPMN patients have multiple lesions in their pancreas, with unique mutations in each lesion [11, 12]. On the other hand, in the results for PDAC patients, we observed samples with only monoclonal mutations (Fig. 5B) and samples with polyclonal mutations (Fig. 5C). Although many PDAC patients typically present with a monoclonal main lesion, recent studies have reported that PanINs surrounding the primary PDAC lesion harbour distinct mutations from the index lesion [6]. The results shown in Fig. 5C likely reflect this genetic heterogeneity in apparently normal pancreases, highlighting the complexity of small background lesions.

**Fig. 6:**
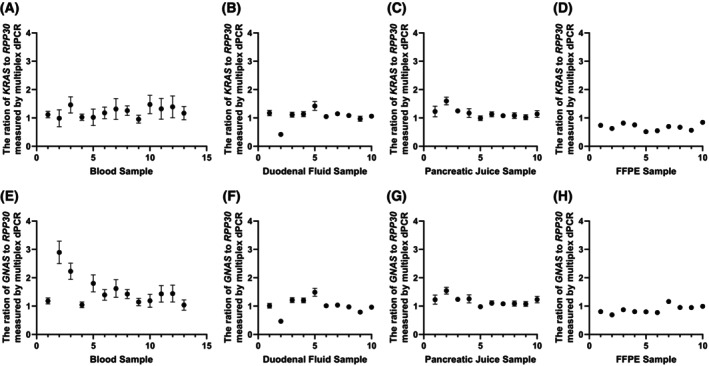
Results of measuring the ratio of the total copy number of *KRAS* or *GNAS* to the copy number of *RPP30* in each clinical sample. Thirteen blood samples; 10 duodenal fluid samples; 10 pancreatic juice samples and 10 FFPE tissue samples were measured via multiplex dPCR combined with melting curve analysis, and the total copy number of the wild‐type and mutant forms of *KRAS* or *GNAS* and the copy number of the reference gene, *RPP30*, were determined. On the basis of the count results, the ratio of the total copy number of *KRAS* or *GNAS* to the copy number of the reference gene *RPP30* was calculated. The error bars indicate the 95% confidence intervals calculated from the Poisson probability. (A)−(D) The ratio of *KRAS* to *RPP30*; (E)−(H) the ratio of *GNAS* to *RPP30*. (A), (E) Blood samples; (B), (F) duodenal fluid samples; (C), (G) pancreatic juice samples; (D), (H) FFPE tissue samples. FFPE, formalin‐fixed, paraffin‐embedded.

Finally, CNA evaluation was performed with DNA obtained from clinical samples. Figure 6 shows the results of measuring the ratio of the total copy number of *KRAS* or *GNAS* to the copy number of *RPP30* in each clinical sample. The error bars indicate the 95% confidence intervals calculated from the Poisson probability. Owing to the low concentration of cfDNA extracted from plasma (257–1756 copies of *RPP30* detected per assay), the error bars are larger than those for other clinical samples. In blood samples #2, #3 and #5, the ratio of the total *GNAS* copy number to the *RPP30* copy number exceeded 1.5 at the lower end of the sampling error, indicating possible *GNAS* gene amplification. In duodenal fluid sample #2, the ratios of the total *KRAS* and total *GNAS* copy numbers to the *RPP30* copy numbers were 0.42 and 0.46, respectively, suggesting possible amplification of *RPP30*, as simultaneous deletion of *KRAS* and *GNAS* is unlikely. In duodenal fluid sample #5 and pancreatic juice sample #2, the ratios of the total *KRAS* copy number and total *GNAS* copy number to the *RPP30* copy number were > 1.4, suggesting possible simultaneous amplification of *KRAS* and *GNAS* or loss of *RPP30*. Re‐evaluation via reference genes other than *RPP30* could clarify the presence or absence of *KRAS* and *GNAS* gene amplification.

## 4 Discussion

In this study, we developed a 14‐plex assay that includes three driver gene hotspots (*KRAS* codon 12/13, *KRAS* codon 61 and *GNAS* codon 201) and a reference gene (*RPP30*). Conventionally, genotyping double‐positive and multi‐positive wells containing multiple genotypes has been challenging in high‐multiplex dPCR. The copy number of the wild‐type allele is greater than that of the low‐frequency mutations. Hence, the number of positive wells dynamically increased with the number of genes for which the wild type was measured, making multiple variant quantification and CNA quantification difficult to perform in a single assay. The developed assay measured genomic DNA standards, and all genotypes were correctly quantified, as shown in Table 1. Furthermore, the LOD of the 10 variants of *KRAS* and *GNAS* was < 0.2%, as shown in Table 2, which is comparable to the LOD of conventional dPCR with a smaller number of multiplexes [30, 31]. These results indicate that multiplex dPCR combined with melting curve analysis has high accuracy in discriminating genotypes in multi‐positive wells and is suitable for assays in which mutant genotyping and CNA quantification are simultaneously performed.

This study demonstrated that genotyping and CNA quantification are possible with all three types of liquid biopsy samples (plasma, duodenal fluid and pancreatic juice) and FFPE tumour tissue samples. In all biopsy samples, mutation frequencies measured by multiplex dPCR correlated with existing methods. The quantitative results of VAF in individual samples revealed that multiple variants were detected in liquid biopsy samples from patients with pancreatic neoplasms, highlighting the field cancerisation which is characteristic of IPMN and, albeit less frequently, PDAC, where polyclonal lesions develop simultaneously in the pancreas [6, 9, 12]. Except a few samples where gene amplification appears to have occurred, the CNA ratios were close to 1, suggesting that the CNAs were measured without being affected by differences in the fragment size distribution of each biopsy sample. As shown in Fig. 6 and Table S3, a few patient samples exhibited indications of *GNAS* gene amplification. Although the error bars are larger than those of the other materials, Blood #2 and #3 displayed significantly larger ratios, suggesting *GNAS* gene amplification. In dPCR, it is well established that measurement results can be influenced by sampling and partitioning errors, which are dependent on the number of copies present in the assay. In Gevensleben et al. [17], *HER2* amplification was measured in plasma cfDNA samples with copy numbers primarily ranging from 300 to 2000. The amplification was evaluated using thresholds based on the number of informative droplets, as determined through the sequential probability ratio test. Similarly, in this study, while plasma samples exhibited larger error bars than other materials due to lower cfDNA concentrations, notable amplification was observed in certain plasma samples. Although no *GNAS* mutations were detected in these samples, such amplification could potentially contribute to pancreatic tumour progression. In the future, the clinical validity of CNA as a biomarker in liquid biopsies from pancreatic cancer patients could be established by concurrently measuring CNA in both liquid biopsies and tumours from the same patient using this multiplex dPCR method.

In Fig. 6, amplification of *RPP30* was observed in a few samples. For example, in duodenal fluid #2, the copy ratios of *KRAS* and *GNAS* were both less than 1, suggesting amplification of the reference gene *RPP30*. It is well established that gene amplification can occur in *RPP30* and *AP3B1*, despite their widespread use as reference genes, as evidenced by data registered in DepMap. While the use of multiple reference genes is effective for achieving more robust measurements, many studies have demonstrated the feasibility of conducting CNA analyses using a single reference, especially under the limited multiplex capabilities of dPCR. For instance, CNA has been measured in a 2‐plex assay by selecting one target gene and one reference gene, such as *HER2*:*EFTUD2* [17] and *HER2*:*RPPH1* [18]. Zhou *et al*. reported constructing four separate 2‐plex dPCR assays, each using *RPP30* as a reference for the CNA quantification of four target genes. Their study revealed that 50% of the mucosal melanomas patients harboured focal amplification of several oncogenes such as *CDK4*, *MDM2* and *AGAP2*, which significantly co‐occurred with amplification of *TERT* [35]. Due to the limited multiplex capabilities of dPCR, researchers have conventionally selected the appropriate reference gene from a pool of well‐characterised candidates through trial and error. This study represents the first demonstration that CNA quantification is feasible even when the number of target genes and their mutants is increased to 10‐plex or more in dPCR. To simultaneously measure multiple mutations and CNAs, *RPP30* was employed as the reference gene. Since dPCR with melting curve analysis supports highly multiplexed assays, the development of robust assays that incorporate multiple reference genes remains a promising area for future exploration.

Among the types of biopsy samples, plasma samples, which can be collected with minimal invasiveness, are the most suitable for disease monitoring. Since the median concentration of cfDNA in the plasma of PDAC patients has been previously reported to be as low as 10 ng·mL^−1^ [15, 29], increasing the feasibility of monitoring plasma samples by increasing the sensitivity of assays analysing these samples is important. We previously reported that false positives occurred in 100% of wild‐type genomic DNA due to PCR errors [29]. We consider that similar false positives also occur in this assay and that using a higher fidelity polymerase will improve sensitivity.

In the examination of LOD, fragmented DNA up to 10 000 copies per assay was used, and the same LOD values were applied to low‐copy clinical samples. Based on our previous research [29], we observed that the LOD of dPCR using melting curve analysis is primarily influenced by PCR errors rather than absolute copy number of the target DNA. Since the LOD remains consistent regardless the number of copies per assay, the same threshold can be applied for determining the presence of target DNA in clinical samples. However, mutant DNA cannot be detected if fewer than one copy is present, meaning that the detection of low‐concentration samples is inherently limited by the original number of mutations present, which directly corresponds to the amount of cfDNA. Several reports have explored the establishment of threshold for low‐copy clinical samples. For instance, the plasmaMATCH study demonstrated the utility of ctDNA testing in advanced breast cancer, employing a threshold of ≥ 300 total copies and ≥ 2 mutant copies to define a positive mutation [36]. Similarly, another study reported that *KRAS* mutations detected in resection or venous margins of PDAC were not predictive of disease recurrence [37]. Given that even healthy individuals can harbour mutations in the *KRAS* gene, the positivity threshold was defined as the mean + 3SD of VAFs observed from healthy samples. Looking ahead, the development of a multiplex dPCR system for pancreatic cancer diagnosis should incorporate a threshold specifically tailored to the unique characteristics of this disease. Such thresholds would enhance the sensitivity and specificity of the assay for detecting clinically relevant mutations in pancreatic cancer.

Molecular diagnostics using liquid biopsy samples are promising tools for patients with many cancer types [38, 39]. However, despite significant efforts to select genotype‐matched therapies, real‐world data suggest limited clinical benefit at high cost [40]. Most cases of PDAC are caused by somatic alterations in four genes, namely, *KRAS*, *CDKNA*, *TP53* and *SMAD4* [41], and alterations in the latter three tumour suppressor genes are crucial for malignant progression. Given their widespread variant distributions, expensive sequencing techniques are required for the mutation profiling of these genes. Oncogenic *KRAS* is considered to emerge at the earliest phase of tumourigenesis, leading to the development of either PanIN or IPMN [8]. IPMNs may also have *GNAS* mutations, which are IPMN‐specific drivers [4]. The presence of *KRAS* and *GNAS* mutations only indicates a predisposition to carcinogenesis and does not induce conversion to invasive carcinoma; however, detecting their molecular diversity associated with field defects may benefit risk stratification for developing invasive tumours, potentially influenced by evolutionary paths [12]. Given that the estimated population prevalence of IPMN is as high as 10.9% [42], cost‐effective IPMN follow‐up methods are needed for early cancer detection and the control of medical costs. By monitoring the changes in VAF balance and CNAs of *KRAS* and *GNAS* via multiplex dPCR, the timing of these changes could be used to monitor the progression of IPMN and other pancreatic cancer precursors.

## 5 Conclusions

Combining melting curve analysis enhances the multiplexing capability of dPCR, allowing for simultaneous detection of multiple genetic alterations. In this study, we developed a 14‐plex assay to simultaneously measure single nucleotide mutations and amplifications of *KRAS* and *GNAS*, which are common driver genes in pancreatic cancer precursors. The amplicons were designed to be approximately the same size, ensuring accurate CNA quantification of fragmented DNA samples. Probes were designed to avoid overlapping probe colours or *T*_m_ values, allowing accurate genotyping of wells containing multiple genotypes. With the 14‐plex assay, genomic DNA derived from cultured cells were successfully measured, confirming accurate genotyping and CNA quantification with a limit of detection below 0.2%. Furthermore, this assay accurately quantified VAFs in liquid biopsy and tissue samples from both pancreatic neoplasm precursor and PDAC patients. Moreover, some patient samples exhibited *GNAS* amplification in the absence of *GNAS* mutations, demonstrating the potential benefits of simultaneously measuring VAF and CNA. These findings highlight the potential of this highly multiplexed dPCR assay for use in cost‐effective patient follow‐up.

## Conflict of interest

JT, TN, YK, TI and TY are employees of Hitachi, Ltd. YO, KT and YM received funding from the Hitachi High‐Tech Corporation (Tokyo, Japan). The other authors declare that they have no competing financial interests.

## Author contributions

JT and TN designed the study. JT, TN and YO performed the experiments, analysed the data and wrote the manuscript. TN and YK developed the instrument for melting curve analysis. YO, HK, KT, HS, ASL and YM provided the clinical samples. TI, TY and YM supervised the study.

## Peer review

The peer review history for this article is available at https://www.webofscience.com/api/gateway/wos/peer‐review/10.1002/1878‐0261.70011.

## Supporting information


**Fig. S1.** Genotyping results of the proposed multiplex assay for each *KRAS* genomic DNA standard.
**Fig. S2.** Genotyping results of the proposed multiplex assay for each *GNAS* genomic DNA standard.
**Fig. S3.** Quantification results for the measured mutant ratio as a function of the input mutant ratio obtained with (A) G12R, (B) G12D, (C) G12V, (D) G12A, (E) G13D, (F) G12S, (G) G12C, (H) Q61H, (I) R201H and (J) R201C fragmented genomic DNA spiked into wild‐type fragmented genomic DNA.
**Fig. S4.** Comparison of *KRAS* and *GNAS* VAFs detected by multiplex dPCR combined with melting curve analysis with *KRAS* and *GNAS* VAFs detected via conventional singleplex dPCR or targeted sequencing, by type of clinical sample.
**Table S1.** List of genomic DNA collected from cell lines used to evaluate CNVs using the multiplex dPCR assay.
**Table S2.** Sequences of primers, probes and a blocker used for multiplex dPCR assays.
**Table S3.** VAFs and CNA ratios of *KRAS* and *GNAS* mutations in clinical samples detected by multiplex dPCR with melting curve analysis.

## Data Availability

The datasets used and analysed during this study are available from the corresponding author upon reasonable request.
