# Supplementary material for: Highly multiplexed digital PCR assay for simultaneous quantification of variant allele frequencies and copy number alterations of KRAS and GNAS in pancreatic cancer precursors
Source: Mol Oncol. 2025 Mar 12;19(10):2921–35. doi: 10.1002/1878-0261.70011 (PMC12515721; doi:10.1002/1878-0261.70011)
Supplement: Supplementary file 1 — Fig. S1. Genotyping results of the proposed multiplex assay for each KRAS genomic DNA standard. Fig. S2. Genotyping results of the proposed multiplex assay for each GNAS genomic DNA standard. Fig. S3. Quantification results for the measured mutant ratio as a function of the input mutant ratio obtained with (A) G12R, (B) G12D, (C) G12V, (D) G12A, (E) G13D, (F) G12S, (G) G12C, (H) Q61H, (I) R201H and (J) R201C fragmented genomic DNA spiked into wild‐type fragmented genomic DNA. Fig. S4. Comparison of KRAS and GNAS VAFs detected by multiplex dPCR combined with melting curve analysis with KRAS and GNAS VAFs detected via conventional singleplex dPCR or targeted sequencing, by type of clinical sample. Table S1. List of genomic DNA collected from cell lines used to evaluate CNVs using the multiplex dPCR assay. Table S2. Sequences of primers, probes and a blocker used for multiplex dPCR assays. Table S3. VAFs and CNA ratios of KRAS and GNAS mutations in clinical samples detected by multiplex dPCR with melting curve analysis. [file MOL2-19-2921-s001.pdf]

Supporting information for

Highly multiplexed digital PCR assay for simultaneous quantification of variant allele frequencies and copy number alterations of *KRAS* and *GNAS* in pancreatic cancer precursors

Junko Tanaka, Tatsuo Nakagawa, Yusuke Ono, Yoshio Kamura, Takeshi Ishida, Hidemasa Kawabata, Kenji Takahashi, Hiroki Sato, Andrew S. Liss, Yusuke Mizukami and Takahide Yokoi

This file includes:

- Table S1
- Table S2
- Table S3
- Fig. S1-1
- Fig. S1-2
- Fig. S2
- Fig. S3
- Fig. S4

Table S1. List of genomic DNA collected from cell lines used to evaluate CNVs using the multiplex dPCR assay.

Cell Line	Format	Product Name	Manufacturer	Description
SW48	Genomic DNA	KRAS Wild Type Reference Standard	Horizon	
RKO	Genomic DNA	EGFR Wild Type Reference Standard	Horizon	
MCF7	Genomic DNA	Genomic DNA - Human Tumor Cell Line: MCF 7	BioChain	
K562	Genomic DNA	K562 Genomic DNA	Promega	
SK-BR-3	Genomic DNA	Genomic DNA Standards for HER2 Measurements	NIST	
MDA-MB-231	Genomic DNA		NIST	
MDA-MB-361	Genomic DNA		NIST	
MDA-MB-453	Genomic DNA		NIST	
BT-474	Genomic DNA		NIST	
BT-20	Genomic DNA	EGFR and MET Gene Copy Number Standards for Cancer Measurements	NIST	
C32	Genomic DNA		NIST	
Daoy	Genomic DNA		NIST	
Hs 746T	Genomic DNA		NIST	
SNU-5	Genomic DNA		NIST	
SW620	Cultured Cell		ATCC	Genomic DNA was extracted using a Blood & Cell Culture DNA Mini Kit (Qiagen).
PC3	Cultured Cell		ATCC	
PC14	Cultured Cell		Riken BRC	
LU99	Cultured Cell		JCRB	

Table S2. Sequences of primers, probes and a blocker used for multiplex dPCR assays. All primers were acquired from Integrated DNA Technologies (Iowa, USA), and all probes were acquired from PentaBase (Odense, Denmark). All probes and the blocker contain 4 hydrophobic DNA analogs that prevent degradation during PCR. Mutation positions are underlined.
dPCR, digital PCR

Primers

Target Gene	Primers	Sequence
KRAS, codon 12/13	Forward Primer	5’-GTCACATTTTCATTATTTTTATTATAAGG-3’
	Reverse Primer	5’-GTCAAGGCACTCTTGCCTAC-3’
KRAS, codon 61	Forward Primer	5’-GATGGAGAAACCTGTCTCTTGGAT-3’
	Reverse Primer	5’-GTCCTCATCTACTGGTCCCTGATTG-3’
GNAS	Forward Primer	5’-GCTTTGGTGAGATCCATTGAC-3’
	Reverse Primer	5’-TCCACCTGGAACTTGGTCTC-3’
RPP30	Forward Primer	5’-ATGGGACTTCAGCATGGCG-3’
	Reverse Primer	5’-CACGGTGAGCGGCTGTCTCC-3’

Probes and blocker for *KRAS*

Target	Description	Sequence	Reporter dye	Quencher
Wild type of codon 12/13	Probe	5'-TTGGAGCTGGTGGCGT-3'	HEX	BHQ-1
G12R	Probe	5'-TTGGAGCT**C**GTGGCGT-3'	ATTO 425	BHQ-1
G12D	Probe	5'-TGGAGCTG**A**TGGCGT-3'	ATTO 425	BHQ-1
G12V	Probe	5'-TGGAGCTG**T**TGGC-3'	ATTO 425	BHQ-1
G12A	Probe	5'-TTGGAGCTG**C**TGGCGT-3'	CAL Fluor Red 610	BHQ-2
G13D	Probe	5'-TTGGAGCTGGTG**A**CGT-3'	FAM	BHQ-1
G12S	Probe	5'-TGGAGCT**A**GTGGCGT-3'	Cy5	BHQ-2
G12C	Probe	5'-GAGCT**T**GTGGCGT-3'	Cy5	BHQ-2
Pseudogene	Blocker	5’-TTGGAGCTGGT**A**GCGT-3’	-	-
Wild type of codon 61/Q61H	Probe	5’-AGGTCA**C**GAGGAGTACAGTG-3’	CAL Fluor Red 610	BHQ-2

Probes for *GNAS*

Target	Description	Sequence	Reporter dye	Quencher
Wild type	Probe	5'-TTCGCTGCCGTGTCC-3'	HEX	BHQ-1
R201H	Probe	5’-TTCGCTGCC**A**TGTCCTG-3’	FAM	BHQ-1
R201C	Probe	5’-TCGCTGC**T**GTGTCC-3’	Cy5	BHQ-2

Probes for *RPP30*

Target	Description	Sequence	Reporter dye	Quencher
Wild type	Probe	5’-ACCTGAAGGCTCTGCGCG-3’	Quasar 705	BHQ-2

Table S3. VAFs and CNA ratios of *KRAS* and *GNAS* mutations in clinical samples detected by multiplex dPCR with melting curve analysis.
VAFs, variant allele frequencies; CNA, copy number alterations.

	#	Disease	VAF										CNA		*RPP30* (Copies)	DNA conc. in liquid samples (ng/mL)
			KRAS								*GNAS*		*KRAS* /*RPP30*	*GNAS* /*RPP30*		
			G12A	G13D	G12R	G12D	G12V	G12S	G12C	Q61H	R201C	R201H				
Blood	1	IPMN /PDAC	0.00%	0.10%	0.00%	0.00%	2.09%	0.00%	0.10%	0.00%	0.10%	0.05%	1.12	1.18	1756	55.8
	2	PDAC	0.00%	0.00%	0.00%	8.63%	0.35%	0.00%	0.00%	0.00%	0.00%	0.12%	0.99	2.89	293	19.9
	3	PDAC	0.00%	0.00%	6.83%	0.00%	0.16%	0.00%	0.00%	0.00%	0.00%	0.00%	1.46	2.23	432	20.1
	4	PDAC	0.00%	0.11%	0.00%	0.06%	0.00%	0.06%	0.00%	0.00%	1.69%	0.62%	1.02	1.04	1702	29.6
	5	IPMN	0.00%	0.00%	0.00%	2.49%	0.00%	0.00%	0.00%	0.00%	0.00%	0.00%	1.02	1.80	315	12.0
	6	IPMN /PDAC	0.00%	0.40%	0.00%	0.13%	1.21%	0.00%	0.40%	0.00%	0.23%	0.00%	1.18	1.40	630	18.1
	7	others: BTC	0.00%	0.00%	0.00%	0.00%	0.29%	0.00%	0.00%	1.37%	0.00%	0.00%	1.31	1.62	261	22.8
	8	IPMN	0.00%	0.09%	0.00%	0.09%	0.00%	0.00%	0.34%	0.00%	0.00%	0.00%	1.26	1.43	930	47.0
	9	PDAC	0.00%	0.00%	0.00%	0.10%	1.87%	0.00%	0.10%	0.00%	0.00%	0.16%	0.96	1.14	1060	42.0
	10	PDAC	0.00%	0.00%	0.00%	0.00%	0.00%	0.19%	0.00%	0.00%	0.00%	0.96%	1.47	1.19	351	23.3
	11	PDAC	0.00%	0.00%	0.00%	0.00%	0.00%	0.28%	0.28%	0.00%	0.00%	0.00%	1.32	1.43	266	26.6
	12	PDAC	0.00%	0.00%	0.00%	0.00%	0.00%	0.00%	0.28%	0.00%	0.00%	3.24%	1.39	1.44	257	23.5
	13	IPMN /PDAC	0.00%	0.17%	0.00%	0.00%	0.00%	0.00%	0.00%	0.00%	3.13%	0.00%	1.17	1.04	493	29.4
Duodenal fluid	1	PDAC	0.00%	0.06%	0.06%	3.64%	0.06%	0.09%	0.03%	0.00%	0.00%	0.00%	1.17	1.01	2735	526.9
	2	IPMN	0.07%	0.04%	0.11%	1.88%	0.22%	0.00%	0.11%	0.09%	0.20%	0.27%	0.42	0.46	6372	368.0
	3	IPMN /PDAC	0.00%	0.06%	0.03%	0.06%	18.99%	0.00%	0.00%	0.00%	0.00%	22.13%	1.12	1.21	3222	929.0
	4	PDAC	0.00%	0.00%	0.06%	19.63%	0.09%	0.06%	0.00%	0.00%	0.08%	0.05%	1.13	1.20	3114	367.9
	5	PDAC	0.06%	0.11%	0.00%	26.05%	0.00%	0.00%	0.00%	0.00%	0.11%	0.05%	1.42	1.49	1230	73.1
	6	PDAC	0.00%	0.00%	0.00%	0.02%	1.30%	0.00%	0.04%	0.00%	0.01%	0.02%	1.05	1.01	12540	1486.5
	7	IPMN	0.00%	0.00%	0.00%	0.04%	0.01%	0.00%	0.03%	0.02%	0.92%	0.07%	1.15	1.03	5864	371.4
	8	IPMN	0.00%	0.07%	0.00%	0.03%	2.05%	0.00%	0.09%	0.00%	2.57%	0.00%	1.08	0.97	5278	617.3
	9	PDAC	0.00%	0.04%	0.00%	0.04%	0.70%	0.04%	0.08%	0.00%	0.10%	0.10%	0.97	0.79	2498	91.5
	10	IPMN /PDAC	0.00%	0.02%	0.00%	0.29%	0.01%	0.00%	0.05%	0.00%	0.05%	0.23%	1.06	0.96	22686	550.0
Pancreatic juice	1	PDAC	0.00%	0.00%	1.60%	1.07%	1.71%	0.00%	0.00%	0.00%	0.00%	0.21%	1.14	1.07	762	3420.0
	2	PDAC	0.00%	0.00%	0.03%	37.95%	0.13%	0.00%	0.00%	0.00%	0.17%	0.00%	1.60	1.54	1858	121.6
	3	IPMN /PDAC	0.07%	0.11%	0.00%	0.05%	34.68%	0.00%	0.00%	0.00%	0.28%	31.30%	1.25	1.24	4881	1733.0
	4	IPMN	0.08%	0.25%	0.42%	29.67%	1.87%	0.00%	0.08%	0.00%	0.48%	0.24%	1.17	1.26	1004	64.7
	5	IPMN	0.00%	0.03%	0.67%	16.33%	12.22%	0.00%	0.00%	0.04%	0.13%	0.77%	0.99	0.98	3173	160.3
	6	IPMN	0.00%	0.03%	0.00%	15.54%	1.15%	0.00%	0.14%	0.03%	0.03%	17.10%	1.13	1.10	3152	143.8
	7	IPMN	0.02%	0.11%	0.98%	4.99%	18.31%	0.20%	0.07%	0.05%	0.71%	18.46%	1.08	1.08	4158	1018.7
	8	PDAC	0.12%	4.93%	4.36%	7.53%	14.92%	0.00%	0.20%	0.15%	0.32%	0.52%	1.08	1.09	2301	79.7
	9	PDAC	0.00%	0.03%	0.31%	1.68%	27.60%	0.03%	0.27%	0.00%	0.07%	0.00%	1.02	1.08	2855	108.2
	10	IPMN	0.06%	0.00%	0.06%	2.26%	9.76%	0.00%	0.00%	0.00%	0.10%	24.68%	1.14	1.23	1595	436.0
FFPE	1	PDAC	0.02%	0.05%	0.02%	0.03%	11.42%	0.00%	0.02%	0.00%	0.10%	0.12%	0.74	0.81	16951	
	2	PDAC	0.01%	0.02%	0.00%	0.01%	8.88%	0.00%	0.05%	0.00%	0.06%	0.08%	0.63	0.69	28809	
	3	PDAC	0.00%	0.07%	0.23%	0.01%	0.03%	0.01%	0.04%	7.79%	0.09%	0.14%	0.82	0.87	11712	
	4	PDAC	0.00%	0.03%	0.01%	0.01%	5.61%	0.01%	0.03%	0.00%	0.10%	0.04%	0.76	0.81	21142	
	5	PDAC	0.00%	0.00%	10.10%	0.02%	0.00%	0.00%	0.10%	0.00%	0.11%	0.16%	0.51	0.80	11547	
	6	PDAC	0.00%	0.02%	19.95%	0.06%	0.01%	0.00%	0.02%	0.00%	0.05%	0.06%	0.55	0.77	39444	
	7	IPMN /PDAC	0.03%	0.03%	0.00%	0.02%	22.45%	0.00%	0.07%	0.00%	0.08%	0.09%	0.70	1.16	14866	
	8	PDAC	0.00%	0.03%	0.00%	5.06%	0.00%	0.00%	0.01%	0.00%	0.08%	0.07%	0.67	0.95	10233	
	9	IPMN	0.00%	0.04%	0.13%	37.84%	0.07%	0.02%	0.02%	0.00%	0.02%	47.22%	0.57	0.95	9525	
	10	IPMN	0.00%	0.02%	0.01%	8.54%	0.03%	0.00%	0.02%	0.00%	0.05%	8.32%	0.85	0.99	11625	

IPMN/PDAC : IPMN with associated PDAC
BTC : Biliary Tract Cancer

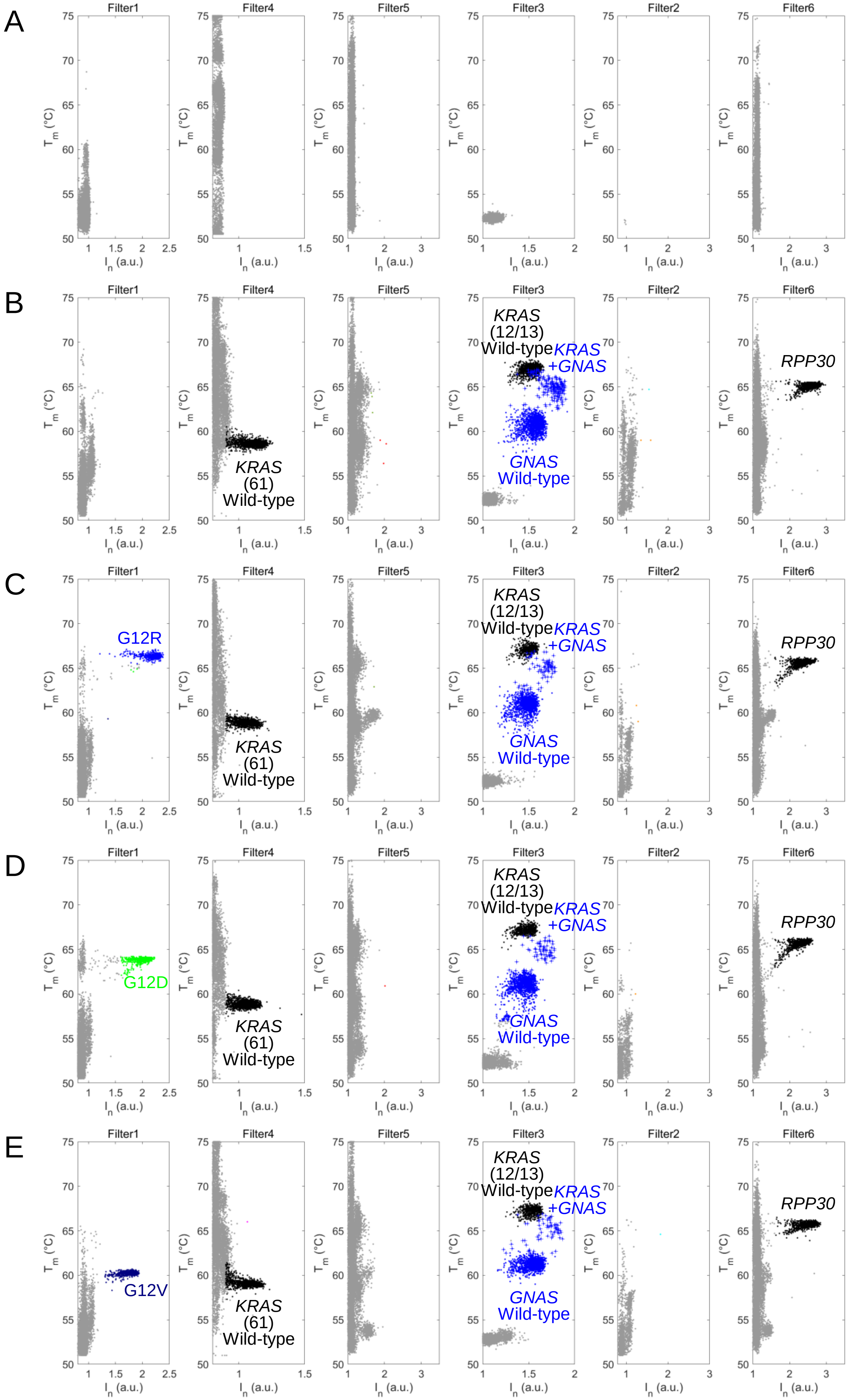

Fig. S1-1. Genotyping results of the proposed multiplex assay for each *KRAS* genomic DNA standard. (A) No template control, (B) Wild type, (C) 50% G12R, (D) 50% G12D, (E) 50% G12V, (F) 50% G12A, (G) 50% G13D, (H) 50% G12S, (I) 50% G12C, and (J) 5% G12D, 25% G13D, 5% G12C and 5% Q61H. Dots indicate a positive well for the wild type or mutant; plus signs indicate a double-positive well for the wild-type of *KRAS* codon 12/13 and *GNAS*. I_n, normalized intensity; T_m, melting temperature.

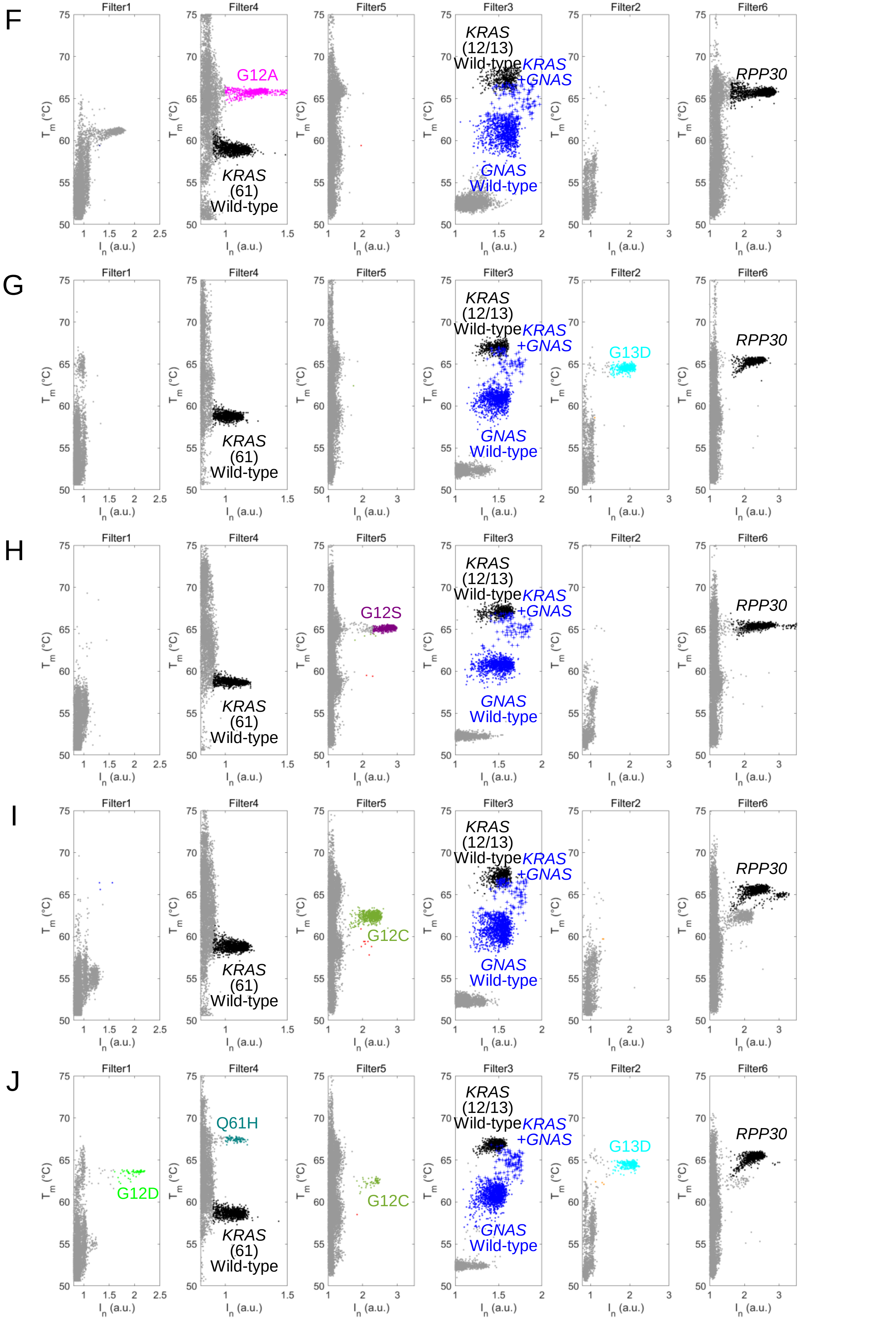

Fig. S1-2. Genotyping results of the proposed multiplex assay for each *KRAS* genomic DNA standard. (A) No template control, (B) Wild type, (C) 50% G12R, (D) 50% G12D, (E) 50% G12V, (F) 50% G12A, (G) 50% G13D, (H) 50% G12S, (I) 50% G12C, and (J) 5% G12D, 25% G13D, 5% G12C and 5% Q61H. Dots indicate a positive well for the wild type or mutant; plus signs indicate a double-positive well for the wild-type of *KRAS* codon 12/13 and *GNAS*. I_n, normalized intensity; T_m, melting temperature.

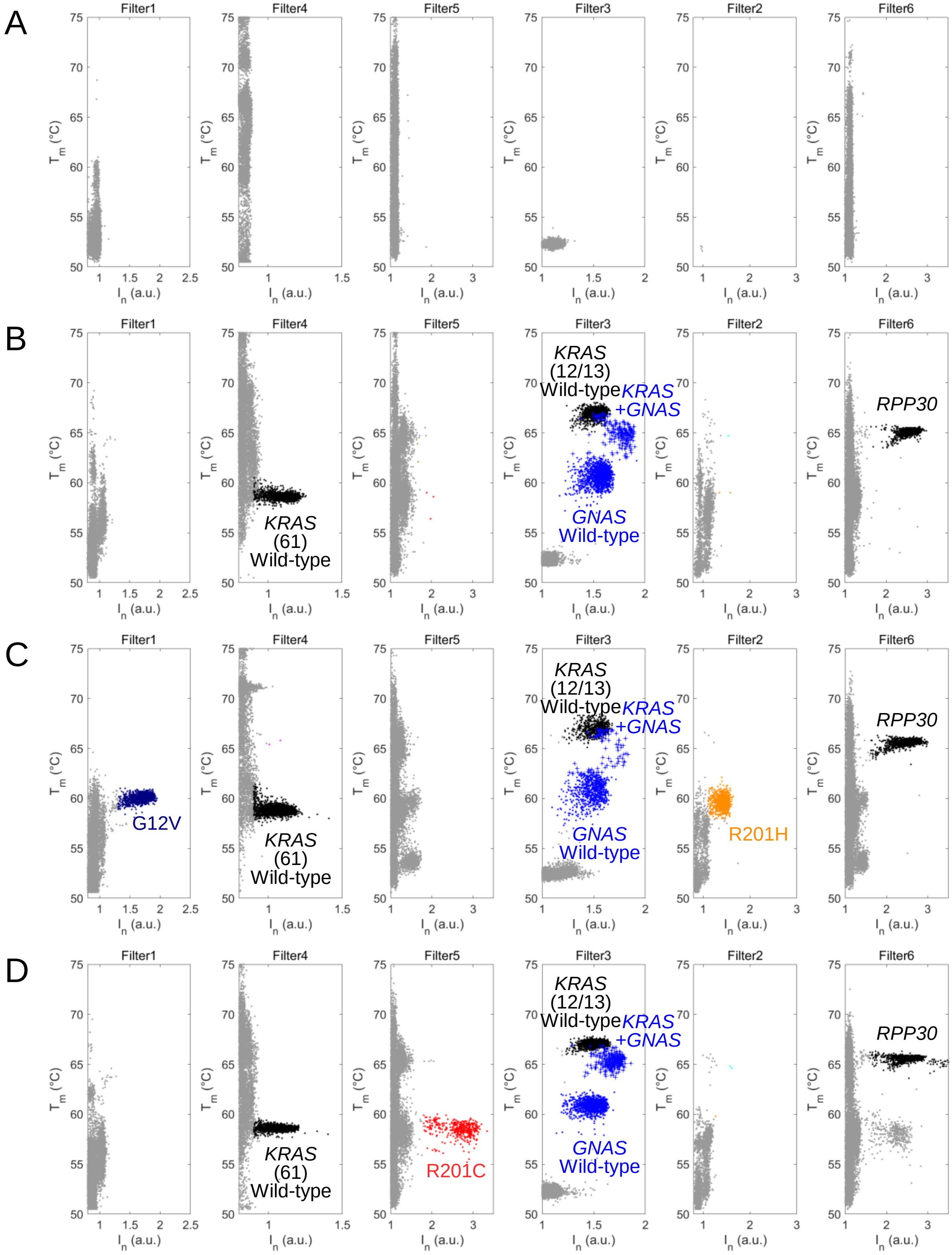

Fig. S2. Genotyping results of the proposed multiplex assay for each *GNAS* genomic DNA standard. (A) No template control, (B) Wild type, (C) 65% *KRAS* G12V and 50% *GNAS* R201H, and (D) 16.3% GNAS R201C. Dots indicate a positive well for the wild type or mutant; plus signs indicate a double-positive well for the wild type of *KRAS* codon 12/13 and *GNAS*. I_n, normalized intensity; T_m, melting temperature.

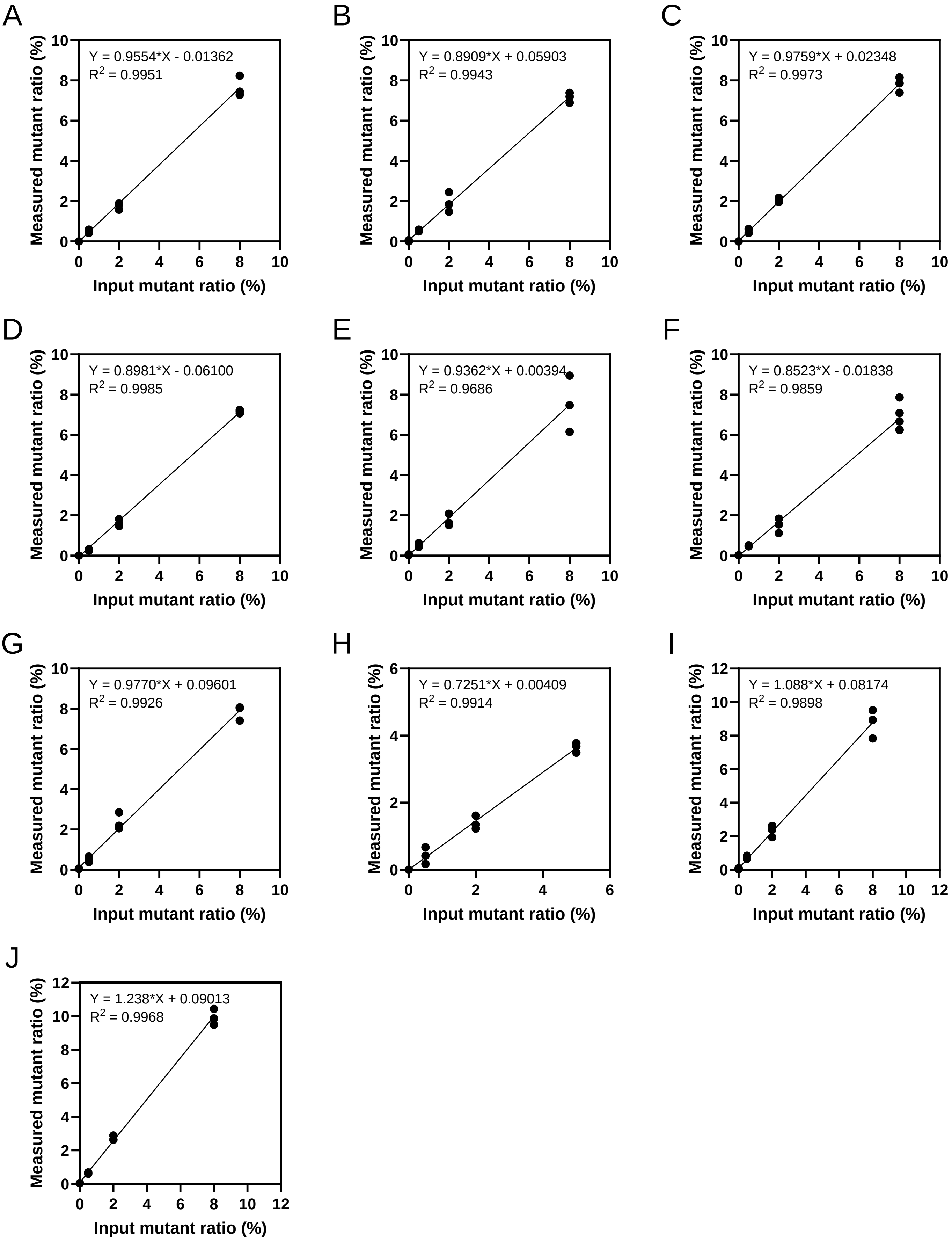

Fig. S3. Quantification results for the measured mutant ratio as a function of the input mutant ratio obtained with (A) G12R, (B) G12D, (C) G12V, (D) G12A, (E) G13D, (F) G12S, (G) G12C, (H) Q61H, (I) R201H and (J) R201C fragmented genomic DNA spiked into wild-type fragmented genomic DNA. N≥3 for each input mutant ratio.

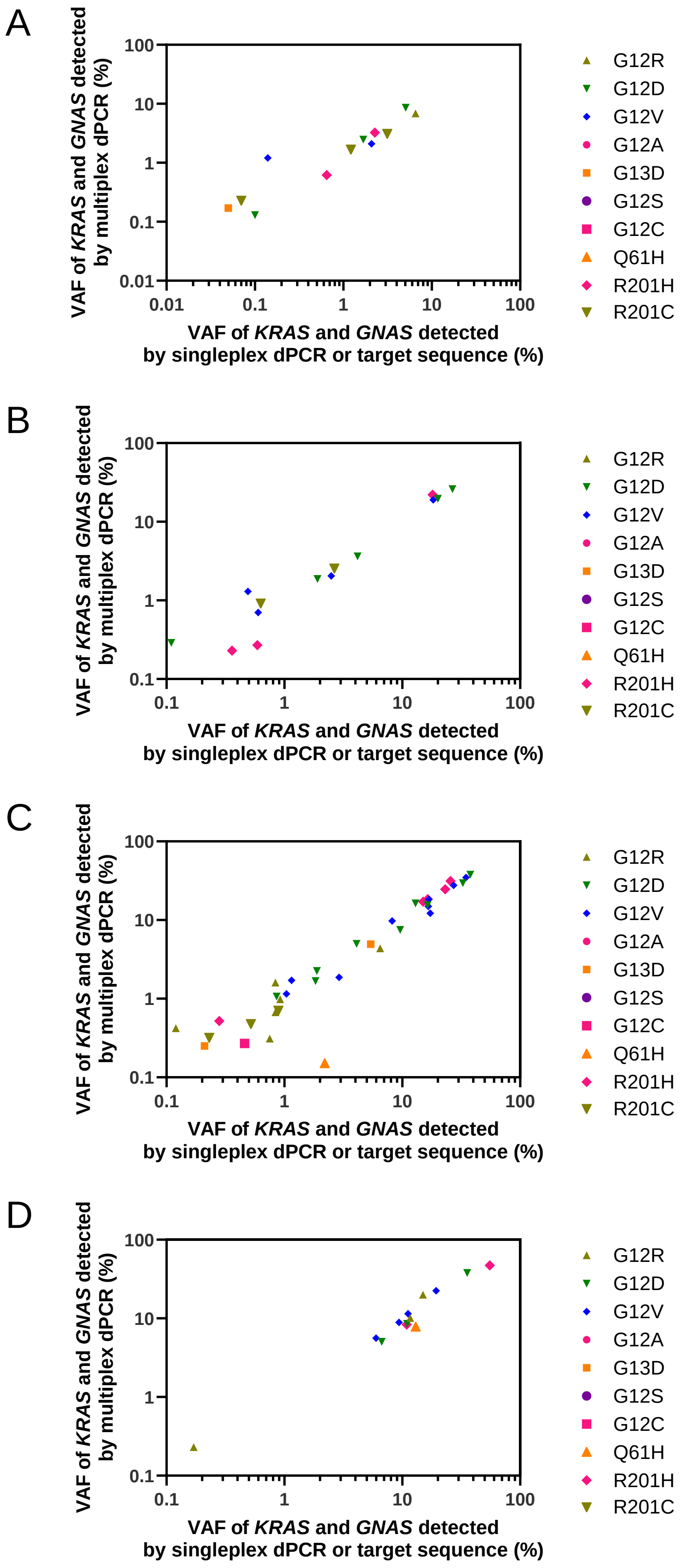

Fig. S4. Comparison of *KRAS* and *GNAS* VAFs detected by multiplex dPCR combined with melting curve analysis with *KRAS* and *GNAS* VAFs detected via conventional singleplex dPCR or targeted sequencing, by type of clinical sample. The VAF of *KRAS* and *GNAS* mutations in all clinical sample types correlated between multiplex dPCR and conventional methods. (A) Blood, (B) duodenal fluid, (C) pancreatic juice and (D) FFPE. VAFs, variant allele frequencies; dPCR, digital PCR; FFPE, formalin-fixed, paraffin-embedded.
